# Psychometric properties of the Chinese (Cantonese) version of the Upper Extremity Functional Index in people with chronic stroke

**DOI:** 10.3389/fneur.2023.989403

**Published:** 2023-02-24

**Authors:** Hong Pan, Shamay S. M. Ng, Tai Wa Liu, Joshua Tsoh, Thomson W. L. Wong

**Affiliations:** ^1^Department of Rehabilitation Sciences, The Hong Kong Polytechnic University, Hong Kong, Hong Kong SAR, China; ^2^School of Nursing and Health Studies, Hong Kong Metropolitan University, Hong Kong, Hong Kong SAR, China; ^3^Department of Psychiatry, Prince of Wales Hospital and Shatin Hospital, Hong Kong, Hong Kong SAR, China

**Keywords:** psychometrics, stroke, rehabilitation, upper extremity, Upper Extremity Functional Index

## Abstract

**Objective:**

To culturally adapt and examine the psychometric properties of the Chinese (Cantonese) version of the Upper Extremity Functional Index (C-UEFI) in people with chronic stroke.

**Design:**

Cross-sectional study.

**Settings:**

University-affiliated neurorehabilitation research laboratory.

**Participants:**

The participants (*N* = 151) were people with chronic stroke (*N* = 101) and healthy controls (*n* = 50).

**Main outcome measures:**

We assessed the C-UEFI, Fugl-Meyer Assessment for Upper Extremity (FMA-UE), Wolf Motor Function Test (WMFT), Six-Minute Walk Test (6MWT), Motor Activity Log (MAL), Activity-Specific Balance Confidence (ABC) scale, Lawton Instrumental Activities of Daily Living (IADL) scale, Survey of Activities and Fear of Falling in the Elderly (SAFFE), Stroke Impact Scale (SIS) and Community Integration Measure (CIM) as outcome measures.

**Results:**

The C-UEFI items demonstrated good test–retest reliability (intraclass correlation coefficient [ICC]_3, 1_ = 0.872) and excellent internal consistency (Cronbach's α = 0.922). People with chronic stroke had poorer C-UEFI scores than the healthy controls. The overall C-UEFI mean score of 101 people with stroke was significantly correlated with the mean scores of the FMA-UE, WMFT, MAL, ABC scale, IADL scale, SAFFE, SIS and CIM and the distance covered in the 6MWT. The C-UEFI cut-off score to distinguish between people with chronic stroke and healthy older adults according to upper extremity function was 57.5 out of 59 (sensitivity: 88.1%; specificity: 84%). The C-UEFI had good content validity, with an acceptable fit to the two-factor structure model.

**Conclusions:**

The C-UEFI is reliable and valid for assessing functional recovery of upper extremity activity in Chinese people with chronic stroke.

## Introduction

Stroke is a common disability in adults, with an estimated 9.6 million ischaemic strokes and 4.1 million haemorrhagic strokes occurring globally each year (1), representing a large economic burden (2). Approximately 80–85% of people with stroke have some degree of upper extremity sensorimotor impairment (3, 4), such as loss of motor control and sensory and proprioceptive deficits (5). Such impairment can directly impact upper extremity-related activities of daily living (ADLs) and social participation in people with stroke (6).

Compared with observational and objective outcome measures, patient-reported outcome measures (PROMs) provide additional value and unique insights into motor recovery after stroke (7). PROMs have been used to screen, monitor progress, and facilitate patient-centered care by gaining insights into patients' views of their physical symptoms, functional abilities and overall psychosocial wellbeing related to their health status (7, 8). Moreover, PROMs can provide data to elucidate the differential effects of interventions based on patients' perspectives. These data can be analyzed to inform rehabilitation programme design to address patients' needs and preferences and improve the quality and efficiency of stroke rehabilitation (9–11). Thus, clinicians need a reliable and valid region-specific PROM that can be used to establish patients' paretic upper limb functions at baseline and monitor patients' progress as a result of treatment.

There are several existing PROMs commonly used to assess the upper limb function of people with stroke, e.g., the Disabilities of the Arm, Shoulder, and Hand (DASH) questionnaire (12), the Stroke Impact Scale (SIS) (13), the Motor Activity Log (MAL) (14), and the Dexterity Questionnaire-24 (DextQ-24) (15). However, these PROMs have limited abilities to capture region-specific upper extremity function (12–15). For example, the DASH questionnaire has been questioned for containing different factors in the contents as the body functions are included (e.g., arm, shoulder, or hand pain) (12, 16). The SIS contains some items unrelated to upper limb-specific tasks, including mood-, language- and memory-related ADL items (13, 16). The MAL is time-consuming because it contains 30 items to assess the amount of use of the more-affected arm and the quality of movement during functional activities (7). The DextQ-24 has 12 out of 24 items evaluating the unimanual upper limb movements of the paretic side in people with chronic stroke and not focus on assessing the upper limb function bilaterally that is particularly important when performing activities of daily living in real-life situations (15).

The Upper Extremity Functional Index (UEFI) is a 20-item, region-specific PROM initially designed to assess upper extremity function in people with musculoskeletal disorders (17, 18). A 15-item version of the UEFI was later developed to increase the reliability and validity as a single-construct interval-level measure of upper extremity function in individuals with upper extremity musculoskeletal disorders (19). The original (English) version of the 15-item UEFI has demonstrated excellent test–retest reliability (intraclass correlation coefficient [ICC]_2, 1_ = 0.95, ICC = 0.94) and excellent internal consistency (person separation index = 0.94) in people with upper extremity musculoskeletal disorders (17, 19).

The UEFI could serve as a region-specific PROM to assess upper extremity function in people with stroke. The UEFI has been translated into several languages for different patient populations. For example, the English (17, 20), Arabic (21), and Turkey (22) versions have been used to assess upper extremity musculoskeletal disorders, the English version has been used for breast cancer (23), and the Arabic version has been used for chronic obstructive pulmonary disease (24). However, the UEFI has not been translated into Chinese, and its psychometric properties have not been examined in Chinese people with stroke. Additionally, the correlations between the 15-item UEFI mean scores and other stroke-related outcome measures have not been evaluated. Thus, the objectives of the current study were to translate and culturally adapt the 15-item UEFI to develop a Chinese (Cantonese) version (C-UEFI) and evaluate the psychometric properties of the C-UEFI for use in assessing upper extremity functional recovery among community-dwelling people with chronic stroke in Hong Kong.

## Materials and methods

### Study design

This study was conducted from January to June 2021 in accordance with the Guidelines for Reporting Reliability and Agreement Studies (25) and the Declaration of Helsinki. Ethical approval was obtained from the Departmental Research Committee of the Hong Kong Polytechnic University (Approval number: HSEARS20210110002). All participants provided written informed consent to participate in this study.

### Translation and cross-cultural adaptation

**Figure 1:**
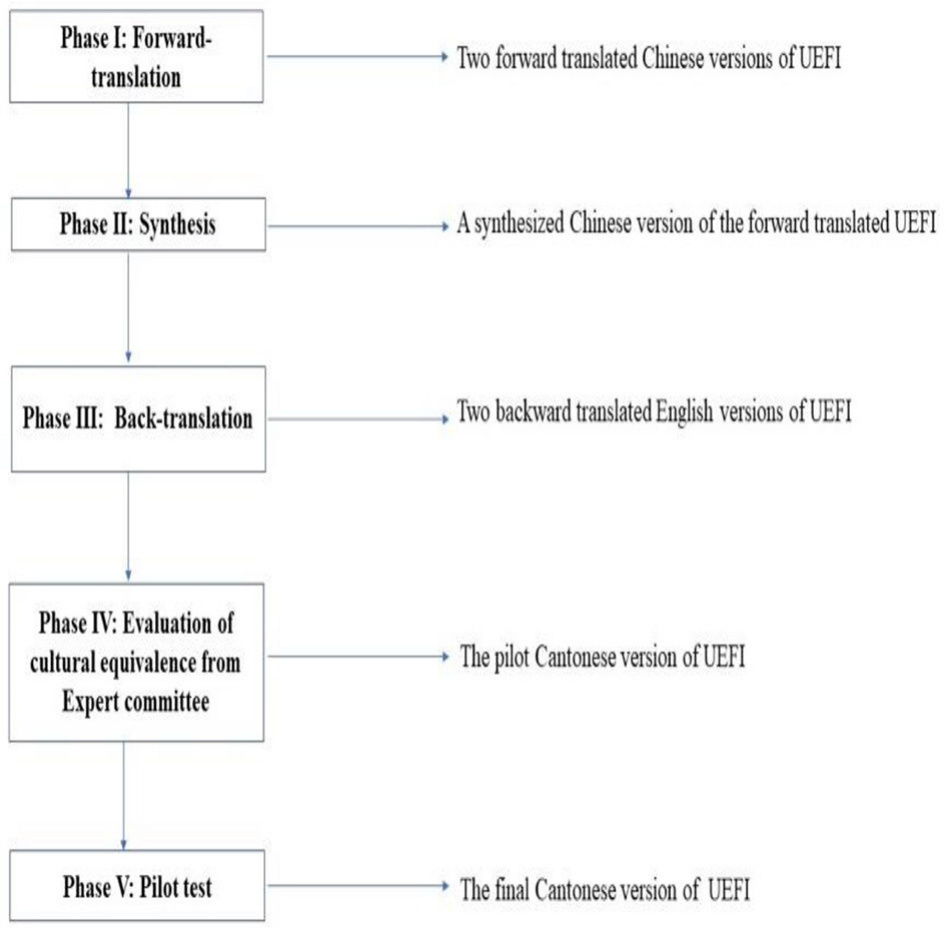
Steps of UEFI translation and cross-cultural adaptation process.

The permission to translate the original English UEFI into Chinese was obtained from the authors of the UEFI. Forward and backward translation and cross-cultural adaptation of the UEFI were performed in accordance with the international guidelines proposed by Beaton et al. (26), which comprise five steps (Figure 1). A panel of six experts was assembled, including two physiotherapists with more than 15 years of clinical experience in stroke rehabilitation, two nurses with more than 10 years of clinical experience and two healthcare professionals. The expert panel rated the experiential, conceptual, semantic, and idiomatic equivalence of each UEFI item using a 4-point Likert scale rating: 1 = not relevant, 2 = somewhat relevant, 3 = quite relevant and 4 = highly relevant. Ratings of 3 or 4 were dichotomised as relevant and 1 or 2 as irrelevant. Among the 15 items, item 7 of the UEFI (driving) was modified into “use of upper limbs in manipulating the shopping cart to buy commodities in the store” for the C-UEFI because it is uncommon for people living in Hong Kong to own a private vehicle.

The pilot C-UEFI was then produced and tested on 10 people with chronic stroke and five healthy controls. All 15 pilot trial participants agreed that the pilot version was fluent, clear, and comprehensible. After the pilot study, no further revision was needed, and the final C-UEFI was established. Finally, we tested the psychometric properties of the C-UEFI.

### Setting and sampling

We recruited 101 people with stroke (58 male, 43 female) from a local self-help group *via* poster advertisements. People with stroke were included in the study if they: (1) were 50–80 years old; (2) suffered a single stroke that was confirmed by magnetic resonance imaging or computed tomography at least 1 year before the start of the study; (3) scored 7 or higher in the Chinese version of the Abbreviated Mental Test (27); (4) could speak Chinese (Cantonese); (5) had volitional control of their non-paretic arm; (6) could induce at least minimal anti-gravity movement in the shoulder of their paretic arm; (7) had at least 5° of wrist extension in the anti-gravity position; and (8) could walk independently for at least 10 m with or without an assistive device. People with chronic stroke were excluded if they: (1) had any other unstable medical conditions (e.g., angina pectoris, pain, or arthritis) or other conditions with medications that may intervene the upper limb function (e.g., Parkinson's disease or Multiple Sclerosis); and (2) had any aphasia or hearing impairment that would affect the data collection procedure.

We recruited 50 healthy older adults (14 male, 36 female) aged 50–80 years with stable health as the control group. People with any comorbid neurological, cardiovascular, or musculoskeletal disease that might affect the assessment were excluded.

The 15-item UEFI has demonstrated excellent test–retest reliability (ICC_2, 1_ = 0.95) in people with upper extremity musculoskeletal disorders (19). However, its reliability in people with stroke has not been evaluated. Assuming that an ICC value for assessing test–retest reliability of the 15-item UEFI in people with stroke was 0.9, a sample size of ≥46 subjects was required to achieve 80% power to detect an ICC of 0.9 with a null hypothesis ICC of 0.8 and a significance level of 0.05. To evaluate the ability of the C-UEFI to discern differences between different groups, ≥50 people with chronic stroke and ≥50 healthy controls were required with a CA_0_ value of 0.3 and CA_1_ value of 0.7 (28). A sample of >100 people with chronic stroke were regarded as reasonable based on the exploratory factor analysis (EFA) estimation (29). Thus, 101 people with stroke were recruited for this study.

### Data collection

All assessments were performed in a university-affiliated neurorehabilitation laboratory. The study objectives and assessment procedures were explained to the participants. After obtaining informed consent, the participants completed a demographic data extraction form.

On day 1, the demographic data of the chronic stroke group (*N* = 101) were collected, and the group completed the C-UEFI. The participants also completed the Fugl-Meyer Assessment of Upper Extremity (FMA-UE), Wolf Motor Function Test (WMFT), Six-Minute Walk Test (6MWT), MAL, Activity-Specific Balance Confidence (ABC) scale, IADL scale, Survey of Activities and Fear of Falling in the Elderly (SAFFE), SIS and Community Integration Measure (CIM) in a randomized order. At least 5 min of rest was allowed between each assessment. After a 7-day interval (day 2), 50 people with chronic stroke were randomly selected from the 101 participants who completed the day 1 assessment, to complete the C-UEFI again to assess test–retest reliability by the same rater who conducted the assessment in day 1.

The healthy control group only completed the C-UEFI on day 1, and their data were used to determine the C-UEFI cut-off scores for people with stroke.

### Outcome measures

#### Upper Extremity Functional Index

The 15-item UEFI retains the rating scale of the 20-item UEFI for all items except item 9 “doing up buttons,” which was modified to a scale from 0 to 3 points based on the Rasch analysis (17). The lowest anchor of item 9, extreme difficulty or unable to perform activity, has the same weight as the other items (=0), but the next two response options are equally weighted: quite a bit of difficulty (=1) and moderate difficulty (=1). The last two response options are weighted as follows: a little bit of difficulty (=2) and no difficulty (=3). All other items are scored using a 5-point adjectival response scale to rate difficulty in performing upper extremity activities: 0 = extreme difficulty or unable to perform activity, 1 = quite a bit of difficulty, 2 = moderate difficulty, 3 = a little bit of difficulty, and 4 = no difficulty.

#### Fugl-Meyer Assessment of Upper Extremity

The FMA-UE is used to evaluate upper limb motor function impairment in people with stroke (30). The FMA-UE comprises 33 items measuring the reflex, movement and coordination of the shoulder, elbow, forearm, wrist, and hand (30). Each item is scored on a 3-point scale from 0 to 2, with a maximum possible score of 66 (30). A higher score indicates a lower level of motor impairment (30). The FMA-UE has demonstrated excellent intra-rater reliability (ICC = 0.995–0.996) and inter-rater reliability (ICC = 0.97) in people with chronic stroke (30, 31).

#### Wolf Motor Function Test

The WMFT is used to assess upper extremity motor function after stroke (32). The scale includes 17 items, comprising 15 function-based tasks and two strength-based tasks. The WMFT yields three scores: a functional ability score, a time score, which quantifies the speed of performance in seconds, and a grip strength score (32). Only the functional ability score was used for the correlation analysis. The functional ability score is rated using a 6-point ordinal scale on 15 items, with a maximum score of 75. A score of 0 indicates no attempt to use the more affected upper extremity, and a score of 5 indicates that movement of the affected upper extremity appears normal (32). The WMFT functional ability score has demonstrated good inter-rater reliability (ICC_3, 1_ = 0.93) and internal consistency (Cronbach's α = 0.92) in people with stroke (33).

#### Six-Minute Walk Test

The 6MWT is used to assess walking endurance (34), a significant predictor of community ambulation and integration in people with stroke (35, 36). Participants were instructed to walk back and forth along a 20-m corridor, covering as much distance as possible in 6 min, taking rests as needed. The maximum distance covered was recorded (36). The 6MWT has demonstrated good inter-rater reliability (ICC = 0.78), inter-rater reliability (ICC = 0.75) and excellent test–retest reliability (ICC_2, 1_ = 0.98) in people with stroke (35, 36).

#### Motor Activity Log

The MAL is used to assess the amount of use (AOU) and quality of movement (QOM) of the paretic arm and hand during ADLs in patients with chronic stroke (15). For each item of AOU and QOM, the scores range from 0 to 5, with total scores ranging from 0 to 150 (15). The MAL has demonstrated excellent internal consistency for AUO (Cronbach's α = 0.88) and QOM (Cronbach's α = 0.91) (15) and excellent test–retest reliability for AOU (*r* = 0.70–0.85) and QOM (*r* = 0.61–0.71) in people with stroke (15).

#### The Activity-Specific Balance Confidence Scale

The ABC scale, a 16-item questionnaire, is used to assess subjective balance confidence in daily activities (37). The items are rated on a scale from 0 to 100. A score of 0 represents no confidence, and a score of 100 represents complete confidence. The total ABC score is calculated by adding the individual item scores together and dividing by the total number of items (38). The ABC scale has demonstrated good test–retest (ICC = 0.85) and high internal consistency (Cronbach's α = 0.97) in people with chronic stroke (38).

#### The Lawton Instrumental Activities of Daily Living Scale

The Lawton IADL scale (14), a self-reported questionnaire, is used to assess the more complex ADLs necessary for living in a community (39). The instrument is most useful for identifying how a person is functioning at present and assessing improvement or deterioration over time. The IADL scale measures nine function items using a 3-point ordinal scale (0 = unable to do, 1 = with assistance, 2 = independent). The IDAL scale has demonstrated excellent inter-rater reliability (ICC_2, 1_ = 0.99) and test–retest reliability (ICC = 0.9) and good internal consistency (Cronbach's α = 0.86) in older adults (14).

#### The Survey of Activities and Fear of Falling in the Elderly

The SAFFE is used to assess the role of fear of falling in activity restriction (40). Fear of falling during the performance of 11 activities is assessed by asking respondents to rate how worried they are about falling during each activity on a 3-point Likert scale (0 = not at all worried, 1 = a little worried, 2 = somewhat worried and 3 = very worried). The total score is the unweighted sum of the 11 items (range: 0–33), with a higher score indicating more fear (41, 42). The SAFFE has demonstrated good internal consistency reliability (Cronbach's α = 0.95) in Chinese older adults (40, 41).

#### Stroke Impact Scale

The SIS is a self-reported questionnaire used to assess the subjective level of disability and health-related quality of life after stroke (13). The SIS comprises 59 items in eight domains and an extra question on stroke recovery. Each item is rated on a 5-point Likert scale in terms of the difficulty in completing each item. The summative scores are generated for each domain, ranging from 0 to 100. The SIS has demonstrated good test–retest reliability (ICC = 0.7–0.92), except for the emotion domain (ICC = 0.57), and good internal consistency (Cronbach's α = 0.90) in people with stroke (13, 43).

#### Community Integration Measure

The CIM assesses the level of community integration using 10 items rated on a 5-point scale, with the total score ranging from 10 to 50 (44). A higher score indicates a higher level of community integration. The Chinese version of the CIM has demonstrated good test–retest reliability (ICC = 0.84) and internal consistency (Cronbach's α = 0.84) in people with chronic stroke (44).

### Statistical analysis

All data were analyzed using SPSS software version 26.0 (IBM Corp., Armonk, NY, USA). The level of confidence for significance was set as α = 0.05. The Shapiro–Wilk test was used to assess data normality. Independent *t*-tests and Mann–Whitney *U* tests were used to compare between-group differences in the parametric and non-parametric data of demographics and variables of interest, respectively. Statistically significant differences between the expected and observed frequencies in categorical variables were calculated using the chi-square test.

Test-retest reliability was assessed using the ICC_3, 1_. The model was based on the two-way mixed effects model and the single-rater type (45, 46). The reliability was defined as excellent (ICC > 0.90), good (ICC: 0.75–0.90), moderate (ICC: 0.50–0.75) and poor (ICC < 0.50) (46). Internal consistency was evaluated using Cronbach's α coefficient, which was rated as follows: very good (0.90–0.95), good (0.80–0.89), fair (0.70–0.79), weak (0.60–0.69) and unacceptable (<0.60) (47). The standard error of measurement (SEM) relative to the total score was rated as follows: very good (<5%), good (5–10%), doubtful (>10–20%) and negative (>20%) (48). SEM was calculated as standard deviation (SD) × $\sqrt{\;\left(1-r\right)}$ (49).

The minimal detectable change (MDC) was calculated as SD × 1.96 × $\sqrt{2\;\left(1-r\right)}$ (50) at a 95% confidence interval, where SD denotes the standard deviation of the baseline total UEFI score, and *r* denotes the test–retest reliability coefficient. Standardized response mean (SRM) was calculated as the ratio of change from pretest to posttest divided by the standard deviation of the change scores. SRM > 0.8, 0.5–0.8, or 0.2– <0.5 indicate large, moderate, and low responsiveness, respectively (51).

Spearman's ρ and Pearson's correlation analyses were used to detect correlations between outcome variables for non-normally and normally distributed data, respectively. Correlation strength was rated as follows: good to excellent (*r* > 0.75), moderate to good (*r* = 0.50–0.75), fair (*r* = 0.25–0.49) and little to no correlation (*r* < 0.25) (52). In order to confirm whether C-UEFI has the ability to determine the difference in the upper limb functions between the people with stroke with upper extremity activity limitations and health older adults, the known-group validity was applied to compare the C-UEFI scores between the stroke group and healthy group. The independent *t*-tests and Mann–Whitney *U* tests were used for parametric and non-parametric data analysis, respectively.

Both EFA and confirmatory factor analysis (CFA) were used to identify the components of the C-UEFI. Principal component analysis and a scree plot were used to determine the optimal number for factor extraction, and promax rotation was used to enhance the interpretability of the factors. In the EFA, the items with factor loadings >0.3 were considered meaningful (53), and these data were input into the CFA model. The commonly used indices were estimated to fit the model of interest according to the following criteria: the comparative fit index >0.95, the Tucker–Lewis index >0.9, the root mean square error approximation <0.06 and the ratio of chi-square to degrees of freedom (χ^2^/df < 3.0) (54).

A receiver operating characteristics (ROC) curve and the areas under the curve (AUCs) were used to determine the optimal cut-off of C-UEFI and the accuracy of ability of C-UEFI to classify the upper extremity activity limitations in people with stroke. The true positive rate and false positive rate were plotted to generate the receiver operating characteristics (ROC) curve to evaluate the performance of C-UEFI as a classifier to distinguish the upper limb functions between the people with stroke with upper extremity activities limitations and healthy subjects. After number of possible cut-off values obtained, the Youden index analysis was performed to identify the optimal cut-off point of the C-UEFI in distinguishing the level of upper extremity activity limitations in people with stroke and their healthy counterparts (55). Area under the curve (AUC) values of <0.5, 0.5–0.7, 0.7–0.8, 0.8–0.9 and >0.9 indicate no, poor, acceptable, excellent, and outstanding discrimination, respectively (56). Youden's index was used to determine the trade-off between maximizing sensitivity and specificity.

## Results

### The translation and cross-cultural adaptation process

“There were only minor linguistic discrepancies between the translators during the forward-backward translation process and resolved through consensus. Our expert panel confirmed the cultural relevancy and linguistic equivalence of the pilot version of Chinese (Cantonese) version of UEFI. The pilot results demonstrated that the fluency, clarity, and comprehensibility of pilot version was good.”

### Participant characteristics

A total of 151 participants (101 people with chronic stroke and 50 healthy older adults) were recruited for this study. Their mean ages were 63.82 (SD 6.4) and 61.58 (7.56) years, respectively (Table 1).

**Table 1:** Demographics of the people with stroke and the healthy older adults.

**Characteristics**	**Stroke group (*N* = 101)**	**Healthy group (*N* = 50)**	**Test value**	***p***
Age, years	63.82 (6.4)	61.58 (7.56)	*t* (−1.906)	0.06
Sex, M/F, *n*	58/43	14/36		
Body mass index (kg/m^2^)	23.56 (3.95)	21.78 (4.38)	*Z* (−3.23)	0.001
Years since stroke	6.74 ± 4.42			
**Stroke-affected side**, ***n***				
Left	46 (46%)			
Right	55 (54%)			
**Stroke type**, ***n***				
Ischemic	69 (68%)			
Hemorrhagic	32 (32%)			
**Mobility status**, ***n***				
Unaided	29 (28.71%)			
Stick	55 (54.46%)			
SBQ	10 (9.9%)			
LBQ	5 (4.95%)			
Others	2 (1.98%)			
**Characteristics**	**Stroke group (*****N*** = **50)**	**Healthy group (*****N*** = **50)**	**Test value**	***p***
Age, years	63.23 (6.18)	61.58 (7.56)	*t* (1.286)	0.201
Sex, M/F, *n*	22/28	14/36		
Body mass index (kg/m^2^)	22.20 (2.32)	21.78 (4.38)	*Z* (−0.052)	0.96
Years since stroke	4.95 ± 1.77			
**Stroke-affected side**, ***n***				
Center	26 (52%)			
Right	24 (48%)			
**Stroke type**, ***n***				
Ischemic	29 (58%)			
Hemorrhagic	21 (42%)			
**Mobility status**, ***n***				
Stick	13 (26%)			
SBQ	17 (34%)			
LBQ	13 (26%)			
Others	7 (14%)			

M, male; F, female. Data were presented as mean and standard deviation of parametric data, median and interquartile range for nonparametric data, and absolute values and percentage of total sample for categorical variables, respectively.

### Reliability, responsiveness, and validity

The ICC_3, 1_ value is shown in Table 2. The test–retest ICC_3, 1_ for the C-UEFI total score was 0.872 (95% CI: 0.798–0.920, *P* < 0.001). The test–retest ICC_3, 1_ for the C-UEFI items ranged from 0.22 (95% CI: −0.023–0.439, *P* = 0.038) to 0.771 (95% CI: 0.651–0.854, *P* < 0.001). The internal consistency (Cronbach's α) of the C-UEFI was 0.922 (Table 3). The SEM and MDC were 3.6 and 9.98, respectively. The SRM was 0.51 (95% CI: 0.09–0.89).

**Table 2:** Test–retest reliability of C-UEFI (*N* = 50).

**Item**	**ICC_3, 1_**	**Lower**	**Upper**	***p-*** **value**
1.	0.543	0.346	0.694	*P* < 0.001
2.	0.385	0.158	0.574	*P* = 0.001
3.	0.581	0.394	0.722	*P* < 0.001
4.	0.684	0.529	0.794	*P* < 0.001
5.	0.541	0.344	0.692	*P* < 0.001
6.	0.653	0.487	0.773	*P* < 0.001
7.	0.733	0.597	0.828	*P* < 0.001
8.	0.771	0.651	0.854	*P* < 0.001
9.	0.736	0.600	0.830	*P* < 0.001
10.	0.539	0.342	0.691	*P* < 0.001
11.	0.220	−0.023	0.439	*P* = 0.038
12.	0.734	0.597	0.829	*P* < 0.001
13.	0.682	0.527	0.793	*P* < 0.001
14.	0.604	0.423	0.738	*P* < 0.001
15.	0.644	0.476	0.767	*P* < 0.001
TS	0.872	0.798	0.920	*P* < 0.001

TS, total score.

**Table 3:** Internal consistency of C-UEFI (*N* = 50).

**Item**	**Corrected item-total correlation**	**Alpha if item deleted**
1.	0.680	0.916
2.	0.631	0.918
3.	0.485	0.923
4.	0.476	0.922
5.	0.558	0.920
6.	0.658	0.917
7.	0.797	0.913
8.	0.780	0.912
9.	0.707	0.915
10.	0.510	0.921
11.	0.615	0.919
12.	0.794	0.913
13.	0.780	0.912
14.	0.683	0.916
15.	0.586	0.921

Cronbach's α coefficient for the entire C-UEFI is 0.922.

The C-UEFI total scores and item scores of the stroke group and healthy group were compared using the known-group validity. The healthy group demonstrated higher levels of upper extremity functional activity than the stroke group (Table 4).

**Table 4:** Known-group validity of C-UEFI (*N* = 50).

**Group**	**Stroke (*****N*** = **50)**		**Healthy (*****N*** = **50)**		**Mann–Whitney *U***	***Z***	***P*** **-value**
**Items**	**Median (IR)**	**Mean rank**	**Median (IR)**	**Mean rank**			
1	3.0 (2.0)	61.18	4.0 (0.0)	105.94	1,028.00	−6.372	< 0.001
2	3.0 (2.0)	68.56	4.0 (0.0)	91.02	1,774.00	−3.55	< 0.001
3	3.0 (2.0)	64.05	4.0 (1.0)	100.13	1,318.50	−5.089	< 0.001
4	4.0 (0.5)	71.15	4.0 (0.0)	85.80	2,035.00	−2.863	< 0.001
5	4.0 (1.0)	70.48	4.0 (0.0)	87.16	1,967.00	−2.882	0.004
6	3.0 (2.0)	61.43	4.0 (0.0)	105.43	1,053.50	−6.395	< 0.001
7	4.0 (1.0)	67.18	4.0 (0.0)	93.82	1,634.00	−4.514	< 0.001
8	3.0 (2.0)	64.55	4.0 (0.0)	99.12	1,369.00	−5.199	< 0.001
9	3.0 (1.5)	51.88	4.0 (0.0)	124.72	89.00	−10.048	< 0.001
10	3.0 (1.0)	64.51	4.0 (2.0)	99.21	1,364.50	−5.334	< 0.001
11	4.0 (0.0)	70.52	4.0 (0.0)	87.06	1,972.00	−3.385	0.001
12	4.0 (1.0)	65.01	4.0 (0.0)	98.19	1,415.50	−5.180	< 0.001
13	3.0 (2.0)	63.60	4.0 (0.0)	101.04	1,273.00	−5.655	< 0.001
14	3.0 (2.0)	66.41	4.0 (0.0)	95.37	1,556.50	−4.230	< 0.001
15	3.0 (2.0)	58.18	4.0 (0.0)	112.00	725.00	−7.499	< 0.001
TS	48 (15.50)	54.95	60 (1.0)	118.52	399.00	−8.447	< 0.001

IR, interquartile range; TS, total score.

### Correlations with other outcome measures

For our stroke participants, their overall C-UEFI mean score showed significant positive correlations with the FMA-UE, WMFT, ABC scale, MAL, Lawton IADL scale, SIS and CIM mean scores (*r* = 0.217–0.759, *P* < 0.05) and with the distance covered in the 6MWT (*r* = 0.519, *P* < 0.001). Their overall C-UEFI mean score also showed a significant negative moderate correlation with the SAFFE mean score (*r* = −0.551, *P* < 0.001) (Table 5).

**Table 5:** Correlations of performance between C-UEFI and stroke-specific impairments in people with chronic stroke (*N* = 101).

	**C-UEFI**	
	**Correlation coefficient**	***p*** **-value**
**Body structure and function domain**		
Fugl-meyer assessment of upper extremity (FMA-UE)	0.423^*^	<0.001
**Activity domain**		
Wolf motor function test (WMFT)	0.42^*^	<0.001
6-min walk test (6 MWT)	0.519^*^	<0.001
Motor activity log (MAL)		
(1) AOU (affected side)	0.536^*^	<0.001
(2) QOM (affected side)	0.519^*^	<0.001
The activity-specific balance confidence (ABC) scale	0.633^*^	<0.001
The Lawton instrumental activities of daily living (IADL) scale	0.711^*^	<0.001
The survey of activities and fear of falling in the elderly (SAFE)	−0.551^*^	<0.001
**Participation domain**		
Stroke impact scale (SIS)		
(1) Strength	0.337^*^	0.001
(2) Hand function	0.534^*^	<0.001
(3) Mobility	0.569^*^	<0.001
(4) ADLs	0.759^*^	<0.001
(5) Memory	0.165	0.099
(6) Communication	0.217^*^	0.029
(7) Emotion	0.262^*^	0.008
(8) Participation	0.589^*^	<0.001
(9) Stroke recovery	0.696^*^	<0.001
Community integration measure (CIM)	0.333^*^	0.001

* The level of confidence for significance was set as α = 0.05. All correlations are Spearman's rho coefficients.

### Factor analysis

**Figure 2:**
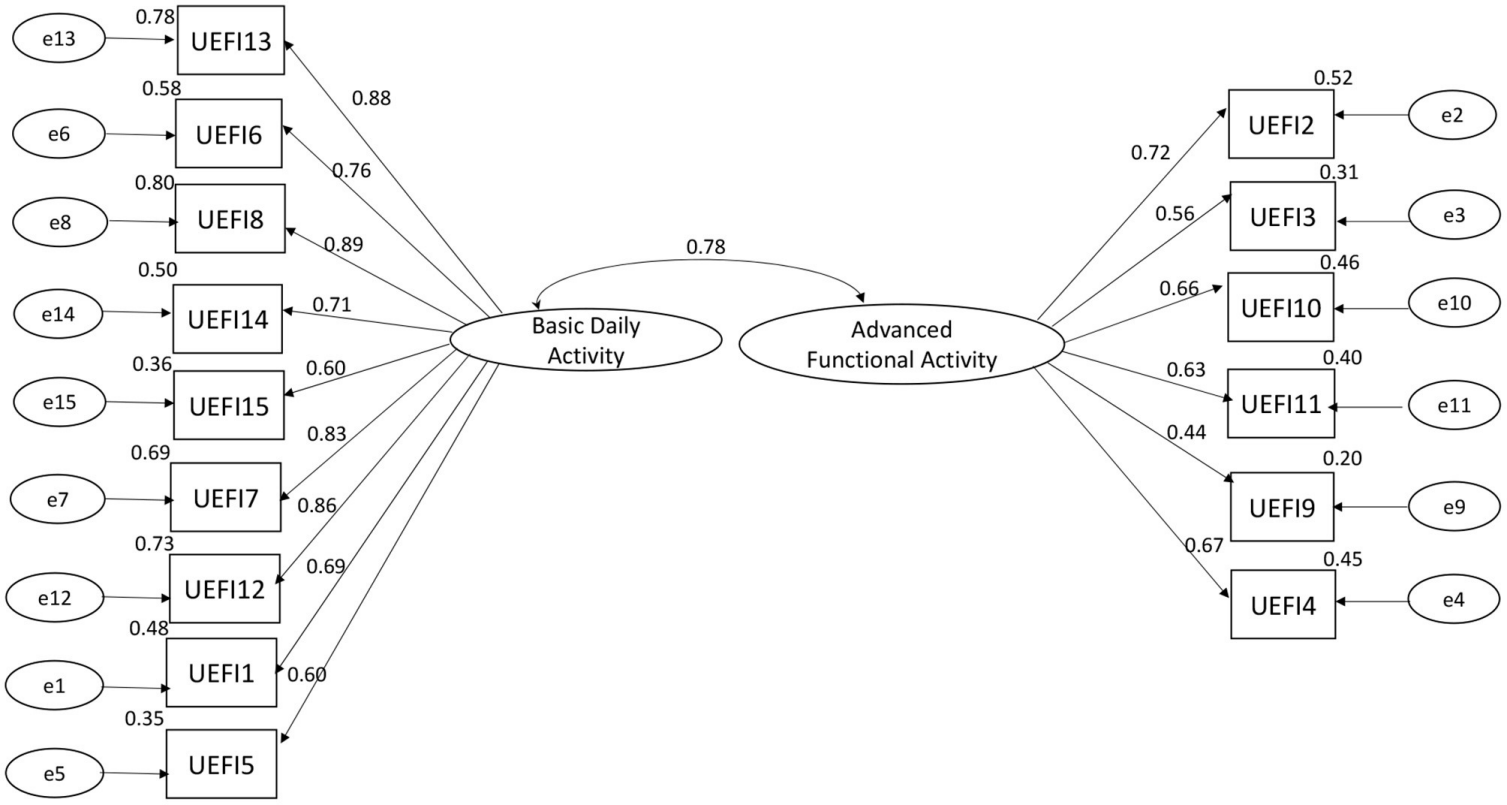
Confirmatory factor analysis of C-UEFI: two factor model (*N* = 101). Rectangles represent the individual items in the Chinese version of the upper extremity functional index. Circles represent measurement errors. Ellipses represent the latent factors of the scale. The data on the double curved line represent the correlation between the two latent factors of the questionnaire. The data on the straight line represent the standardized regression weight. The data beside the rectangle represent the squared multiple correlation.

The Kaiser–Meyer–Olkin measure was 0.886, which indicated sufficient C-UEFI items for the factor analysis. Bartlett's test of sphericity was significant, showing that the factor analysis was satisfactory. The EFA suggested a two-factor model, which explained 58.434% of the total variance (Table 6). The CFA model is presented in Figure 2 and Table 7. Items 13, 6, 8, 14, 15, 7, 12, 1, and 5 were specified to load on the “Basic Daily Activity” factor, and items 2, 3, 10, 11, 9, and 4 were specified to load on the “Advanced Functional Activity” factor. Although some parameters of the CFA did not reach the significance threshold, this model displayed an acceptable fit, with a χ^2^/df of 2.27 (*P* < 0.001), a robust comparative fit index of 0.872, a robust Tucker–Lewis index of 0.849 and a robust root mean square error approximation of 0.113. The inter-factor correlation between the two subscales was significant (*r* = 0.78). The Cronbach's α values for the two subscales (α_1_ = 0.922; α_2_ = 0.774) and the total score (α = 0.921) were acceptable.

**Table 6:** The rotated factor matrix of exploratory factor analysis of the C-UEFI: two factor model (*N* = 101).

**Factors**		
**Item**	**Basic daily activity (one)**	**Advanced functional activity (two)**
13	0.900	
6	0.786	
8	0.767	
14	0.758	
15	0.735	
7	0.705	
12	0.698	
1	0.549	
5	0.507	
2		0.753
3		0.746
10		0.700
11		0.576
4		0.543
9		0.516
Eigenvalues	7.394	1.371
Variance explained (%)	58.434	

**Table 7:** Confirmatory factor analysis and item statistics of the C-UEFI (*N* = 101).

**Items description**	**Regression weight**
**Basic daily activity (one)**	
13. Laundering clothes (e.g., washing, ironing, folding)	0.88
6. Preparing food (e.g., peeling, cutting)	0.76
8. Vacuuming, sweeping, or raking	0.89
14. Opening a jar	0.71
15. Carrying a small suitcase with your affected limb	0.60
7. Using the shopping cart to buy items in the store	0.83
12. Cleaning	0.86
1. Any of your usual work, housework, or school activities	0.69
5. Pushing up on your hands (e.g., from bathtub or chair)	0.60
**Advanced functional activity (two)**	
2. Lifting a bag of groceries to waist level	0.72
3. Placing an object onto, or removing it from, an overhead shelf	0.56
10. Using tools or appliances	0.66
11. Opening doors	0.63
9. Doing up buttons (Note: response numbering is correct)	0.44
4. Washing your hair or scalp	0.67
Robust χ^2^/df	2.27 (*P* < 0.001)
Robust CFI	0.872
Robust TLI	0.849
Robust RMSEA	0.113
Inter-factor correlation	0.78
Cronbach α value for two subscales	0.922 (one)/0.774 (two)
Cronbach α value for total score	0.921

### Distinguishing cut-off score

**Figure 3:**
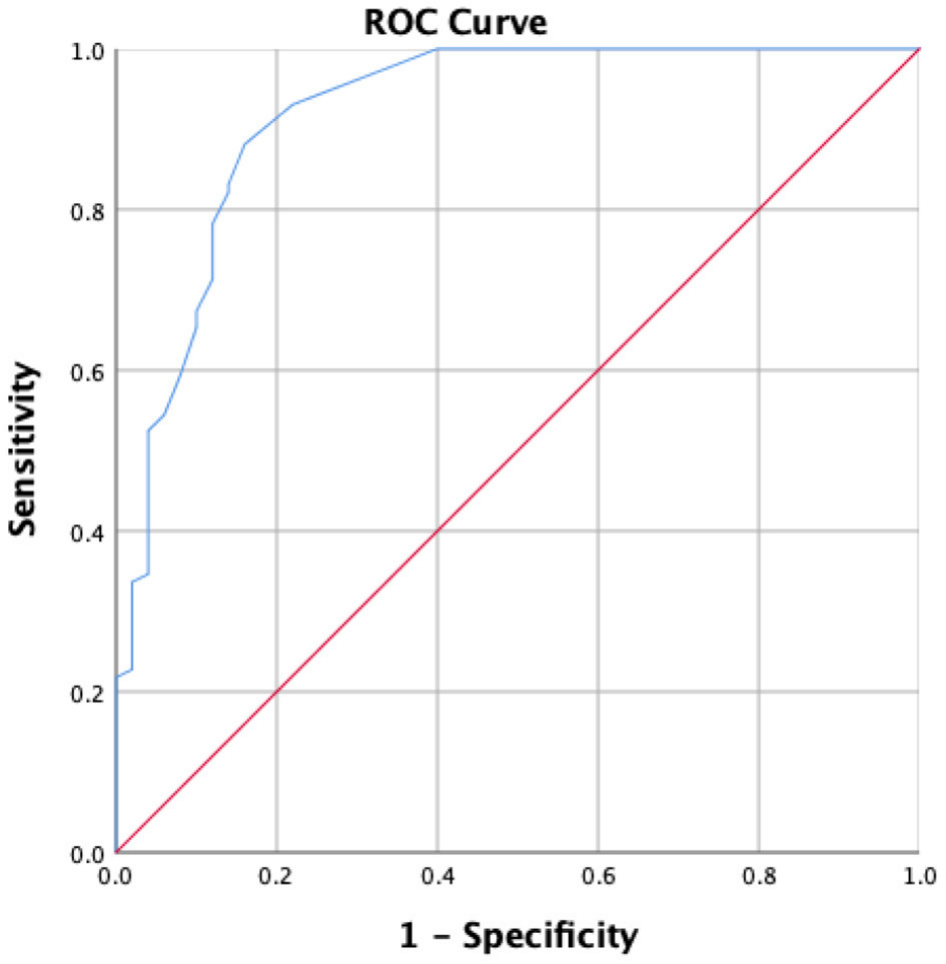
Receiver operator characteristics of the C-UEFI to distinguish stroke subjects (*N* = 101) from healthy subjects (*N* = 50).

A C-UEFI cut-off score of 57.5 was identified. The receiver operating characteristic curve yielded an AUC of 0.921, with a sensitivity of 89.1% and a specificity of 84% of 1.721 of Youden's index, respectively, and with positive predictive value of 91.8% and negative predictive value of 79.2%, separately (Table 8, Figure 3).

**Table 8:** Value of area under the receiver operating characteristic curve, sensitivity, and specificity for the optimal cut-offs of C-UEFI (*N* = 101).

**Comparison**	**Sensitivity (95%CI)**	**Specificity (95%CI)**	**Positive predictive value (95%CI)**	**Negative predictive value (95%CI)**	**AUC**	**Youden Index J**	**Optimal Criterion**
Upper limb impairment vs. no impairment	89.1 (0.83–0.95)	84 (0.74–0.94)	0.918 (0.86–0.97)	0.792 (0.68–0.90)	0.921	1.721	57.5

CI, confidence interval; AUC, area under the receiver operating characteristic curve.

## Discussion

This is the first study to extend the use of the UEFI to assess the region-specific PROM of the level of upper extremity function in community-dwelling people with stroke. In summary, the C-UEFI demonstrated good test–retest reliability and excellent internal consistency in people with chronic stroke. People with stroke scored lower than healthy controls on all items of the C-UEFI. The C-UEFI score was significantly correlated with the scores of FMA-UE, WMFT, ABC scale, MAL, Lawton IADL scale, SAFFE, SIS, and CIM and with the distance covered in the 6MWT. Our study also demonstrated that 57.5 was the optimal cut-off UEFI score to differentiate between people with stroke and healthy older adults according to upper extremity function. A two-factor structure comprising “Basic Daily Activity” and “Advanced Functional Activity” was confirmed by factor analysis.

### UEFI scores in people with chronic stroke

In our study, the PROM of upper extremity function of people with stroke was significantly lower than that of healthy controls on all items of the C-UEFI. Stroke-specific impairments of upper and lower extremities include muscle weakness, spasticity, and impaired motor control (57), which are caused by insufficient motor unit recruitment, reduced muscle motor unit firing rates (58), and poor voluntary activation (59). These impairments may worsen the ability of people with stroke to execute specific ADL movements, resulting in poorer C-UEFI scores compared with healthy older adults.

### UEFI reliability

This was the first study to investigate the reliability of the C-UEFI for assessing people with chronic stroke. The good test–retest reliability (ICC_3, 1_ = 0.872) indicates that the C-UEFI is a reliable outcome measure for clinical use in Chinese people with chronic stroke. This finding is consistent with previous studies showing excellent test–retest reliability of the English version in people with upper extremity musculoskeletal disorders (ICC_2, 1_ = 0.95, ICC = 0.94) (17, 19). Three reasons may explain the good results achieved. First, we recruited people with chronic stroke (post-stroke duration of 6.74 ± 4.42 years), which indicates that the potential functional recovery of their upper extremity activity has plateaued, resulting in low variability in the test–retest performance. Second, our standardized experimental protocol and well-trained raters may have helped minimize measurement error. Third, the 7-day test–retest interval adopted in our study is suitable for minimizing learning effects and preventing changes in the conditions of participants.

The Cronbach's α coefficients were excellent for the individual items (Cronbach's α = 0.912–0.923) and total scores (Cronbach's α = 0.922) of the C-UEFI, indicating good correlations between each item. This finding is consistent with previous studies that reported excellent internal consistency in people with upper extremity musculoskeletal disorders (Cronbach's α = 0.94) (19). The high internal consistency of the C-UEFI may indicate that individual items measured the same concept and domain.

The measurement error of the C-UEFI in people with chronic stroke was quantified in this study using SEM and MDC. The SEM (3.6) represents 6.1% of the C-UEFI score range, indicating that the measurement error of the C-UEFI represents only a small portion of the total scale range. The MDC (9.98) represents 16.9% of the total score range, which is similar to the MDC (8.1) previously reported in people with upper extremity musculoskeletal disorders (19). The MDC indicates that a change of at least 9.98 is required to be considered a true change in the level of upper extremity functional activity in people with chronic stroke. The C-UEFI cut-off score (57.5) distinguishing between people with chronic stroke and healthy older adults in this study markedly surpasses the calculated MDC (9.98). This between-group disparity suggests that the difference was genuine and not caused by measurement error. The SRM of C-UEFI in people with stroke shown a moderate degree responsiveness from two evaluations, which indicates the C-UEFI could be used to detect the people with stroke's upper extremity activity limitations.

### Correlations with other outcome measures

A significant fair correlation was detected between C-UEFI and FMA-UE scores. FMA-UE scores reflect neural motor control, including upper extremity muscle performance and gross gripping function, which are essential components of the level of upper extremity functional activity (60). Hence, it is expected that people with stroke with a high FMA-UE score would achieve a high C-UEFI score.

Expectedly, the C-UEFI score showed significant correlations with WMFT and MAL scores. The WMFT is a quantitative outcome measure of upper extremity motor ability in people with stroke, mainly rating upper extremity strength and dexterity (32), similar to some items of the C-UEFI. The MAL is a semi-structured interview used to assess arm function during 30 daily functional tasks (15). The C-UEFI and MAL items assessing upper extremity functional activities in ADLs are similar. Hence, the C-UEFI and MAL share similar contents and the same ICF construct.

The C-UEFI score showed significant correlations with the distance covered in the 6MWT and the ABC scale scores. The 6MWT assesses the distance walked in 6 min as a sub-maximal test of aerobic capacity or exercise endurance (35). The ABC scale is a self-reported outcome measure of balance confidence in performing various daily activities without losing balance or experiencing a sense of unsteadiness (37). Aerobic capacity, walking endurance and balance performance in daily activities are beneficial for coordinating the upper extremity to execute specific activities in some items of the C-UEFI, e.g., items 2, 3, 7, and 15. Hence, the C-UEFI scores were significantly positively correlated with the distance covered in the 6MWT and the ABC scale scores.

Our study demonstrated that the C-UEFI score showed a significant positive good to excellent correlation with the Lawton IADL scale score. The Lawton IADL scale evaluates the ability to perform complex ADLs necessary for independent living in the community (39), and the C-UEFI evaluates functional activities using the upper extremities in ADLs. Thus, the C-UEFI and Lawton IADL scale share similar contents and the same ICF construct and domain. Our study also demonstrated that the C-UEFI showed a significant negative moderate to good correlation with the SAFFE. The SAFFE was developed as an outcome measure to evaluate avoidance behavior due to fear of falling by quantifying self-perceived and observable ADLs and instrumental ADLs (41). Thus, reduced upper extremity mobility could increase the fear and risk of falling in people with stroke.

The C-UEFI score was significantly correlated with the SIS score, except for the memory evaluation. The SIS is used to evaluate the health-related quality of life in the upper extremity activity domain (13). Higher UEFI scores reflect increased use of the upper limbs in ADLs and thus higher SIS scores. The C-UEFI score was also significantly correlated with the CIM score. CIM is a subjective outcome measure of community integration and considers the self-reported subjective feelings of participants (44). Hence, improving upper extremity mobility could increase participation in daily activities, which could enhance community integration.

### Factor analysis

Our study used factor analysis to explore the components of the C-UEFI. A two-factor structure was identified according to the EFA, namely, “Basic Daily Activity” and “Advanced Functional Activity,” which was confirmed by the CFA. The “Basic Daily Activity” dimension (items 13, 6, 8, 14, 15, 7, 12, 1, and 5) mainly evaluates basic ADLs, which require basic movements. The “Advanced Functional Activity” dimension (items 2, 3, 10, 11, 4, and 9) mainly evaluates instrumental ADLs, which require more complex skills and coordination. The factor analysis revealed the option to split the C-UEFI questionnaire into two parts: basic ADLs and instrumental ADLs. Future studies are needed to provide further evidence on the psychometric properties of the newly derived subscales. Moreover, some items (e.g., 6, 7, 8, and 14) in the “Basic Daily Activity” dimension also involve the application of instruments. Future studies using Rasch analysis are needed to provide a more robust conclusion regarding this two-factor structure model.

### Optimal cut-off score

Our results showed that a UEFI score of 57.5 was sufficiently sensitive for discriminating between people with chronic stroke and healthy older adults (AUC = 0.921; sensitivity = 88.1%; specificity = 84% of Youden's index). The high AUC indicates excellent accuracy of the C-UEFI in discriminating between those two groups. Thus, the C-UEFI is a sensitive and specific test for identifying people with stroke who have upper extremity functional activity limitations.

## Limitations

Several limitations of this study should be acknowledged. First, our participants were recruited from a local self-help group for people with stroke, and those who attend such groups may have a relatively high level of functional mobility. Future studies should include participants with lower levels of functional mobility to increase the generalisability of our results to the stroke population. Second, stroke incidence is associated with age, doubling with each decade after 55 years (61), only people aged ≥ 50 years were recruited in this study. Thus, the results in our study only apply to those participants who fulfilled our inclusion criteria. Future research is needed to verify whether the C-UEFI could be extended to those who are younger. Third, compared with men, women with stroke tend to be more disabled, and there may be sex differences in the levels of upper extremity functional activity (62). Thus, future studies should evaluate differences in the levels of upper extremity functional activity between men and women with chronic stroke.

## Conclusions

The results of this study demonstrate that the C-UEFI is a reliable, valid, sensitive, and specific clinical test for evaluating functional recovery of upper extremity activity in people with chronic stroke.

## Data availability statement

The raw data supporting the conclusions of this article will be made available by the authors, without undue reservation.

## Ethics statement

The studies involving human participants were reviewed and approved by the Departmental Research Committee of The Hong Kong Polytechnic University. The patients/participants provided their written informed consent to participate in this study.

## Author contributions

HP and SN contributed to the conception and design of the study. HP, TL, JT, and TW collected the data and organized the database and wrote the first draft of the manuscript. HP, SN, and TL performed the statistical analysis. All the authors contributed to manuscript revision, read, and approved the submitted version of the manuscript.

## References

[1] BCV Khatri P. Stroke. Lancet. (2020) 396:129–42. 10.1016/S0140-6736(20)31179-X 32653056

[2] Sevick LK Ghali S Hill MD Danthurebandara V Lorenzetti DL Noseworthy T. Systematic review of the cost and cost-effectiveness of rapid endovascular therapy for acute ischemic stroke. Stroke. (2017) 48:2519–26. 10.1161/STROKEAHA.117.017199 28716983

[3] Wattchow KA McDonnell MN Hillier SL. Rehabilitation interventions for upper limb function in the first 4 weeks following stroke: a systematic review and meta-analysis of the evidence. Arch Phys Med Rehabil. (2018) 99:367–82. 10.1016/j.apmr.2017.06.014 28734936

[4] Santisteban L Térémetz M Bleton JP Baron JC Maier MA Lindberg PG. Upper limb outcome measures used in stroke rehabilitation studies: a systematic literature review. PLoS ONE. (2016) 11:e0154792. 10.1371/journal.pone.0154792 27152853 PMC4859525

[5] Pollock A Farmer SE Brady MC Langhorne P Mead GE Mehrholz J. Interventions for improving upper limb function after stroke. Cochrane Database Syst Rev. (2014) 2014:CD010820. 10.1002/14651858.CD010820.pub2 25387001 PMC6469541

[6] Veerbeek JM Kwakkel G van Wegen EE Ket JCF Heymans MW. Early prediction of outcome of activities of daily living after stroke: a systematic review. Stroke. (2011) 42:1482–8. 10.1161/STROKEAHA.110.604090 21474812

[7] Stewart JC Cramer SC. Patient-reported measures provide unique insights into motor function after stroke. Stroke. (2013) 44:1111–6. 10.1161/STROKEAHA.111.674671 23422082 PMC3609884

[8] Moloney J Walshe M Regan J. Patient reported outcome measures in dysphagia research following stroke: a scoping review and qualitative analysis. Dysphagia. (2022) 38:181–90. 10.1007/s00455-022-10448-y 35467246 PMC9873730

[9] van Lieshout EC Visser-Meily JMA Nijland RH Dijkhuizen RM Kwakkel G. Comparison of self-reported vs. observational clinical measures of improvement in upper limb capacity in patients after stroke. J Rehabil Med. (2020) 52:jrm00051. 10.2340/16501977-2661 32179928

[10] Price-Haywood EG Harden-Barrios J Carr C Reddy L Bazzano LA Driel MLV. Patient-reported outcomes in stroke clinical trials 2002–2016: a systematic review. Qual Life Res. (2019) 28:1119–28. 10.1007/s11136-018-2053-7 30465318

[11] Reeves M Lisabeth L Williams L Katzan I Kapral S Deutsch A. Patient-reported outcome measures (proms) for acute stroke: rationale, methods and future directions. Stroke. (2018) 49:1549–56. 10.1161/STROKEAHA.117.018912 29789396

[12] Dalton E Lannin NA Laver K Ross L Ashford S McCluskey A. Validity, reliability and ease of use of the disabilities of arm, shoulder and hand questionnaire in adults following stroke. Disabil Rehabil. (2017) 39:2504–11. 10.1080/09638288.2016.1229364 27767374

[13] Vellone E Savini S Fida R Dickson VV Melkus GD Carod-Artal FJ. Psychometric evaluation of the stroke impact scale. J Cardiovasc Nurs. (2015) 30:229–41. 10.1097/JCN.0000000000000145 24695074

[14] van der Lee JH Beckerman H Knol DL de Vet HCW Bouter LM. Clinimetric properties of the motor activity log for the assessment of arm use in hemiparetic patients. Stroke. (2004) 35:1410–4. 10.1161/01.STR.0000126900.24964.7e 15087552

[15] Dehmiyani A Taghizadeh G Azad A Goudarzi S Jamali S Hejazi Shirmand M. Psychometric properties of dexterity questionnaire-24 in Iranian chronic stroke survivors. Top Stroke Rehabil. (2022) 29:201–7. 10.1080/10749357.2021.1970451 34429044

[16] Hill B Bialocerkowski A Williams G. Do patient reported outcome measures capture actual upper limb recovery? Int J Ther Rehabil. (2014) 21:558–9. 10.12968/ijtr.2014.21.12.558

[17] Chesworth BM Hamilton CB Walton DM Benoit M Blake TA Bredy H. Reliability and validity of two versions of the upper extremity functional index. Physiother Can. (2014) 66:243–53. 10.3138/ptc.2013-45 25125777 PMC4130402

[18] Arumugam V MacDermid JC. Clinimetrics: upper extremity functional index. J Physiother. (2018) 64:125. 10.1016/j.jphys.2018.01.003 29555423

[19] Hamilton CB Chesworth BM. A rasch-validated version of the upper extremity functional index for interval-level measurement of upper extremity function. Phys Ther. (2013) 93:1507–19. 10.2522/ptj.20130041 23813086 PMC3827714

[20] Hadadi M Ebrahimi S Sarvestani M Mozafari M. Correspondence: upper extremity functional index. J Physiother. (2020) 66:278. 10.1016/j.jphys.2020.09.004 32988792

[21] Aljathlani MF Alshammari MO Alsuwaygh MA Al-Mutairi M Aljassir FF Bindawas SM. Cross-cultural adaptation and validation of the Arabic version of the upper extremity functional index. Disabil Rehabil. (2021) 38:1–7. 10.1080/09638288.2021.1947396 34227453

[22] Aytar A Yuruk ZO Tuzun EH Baltaci G Karatas M Eker L. The upper extremity functional index (uefi): cross-cultural adaptation, reliability, and validity of the turkish version. J Back Musculoskelet Rehabil. (2015) 28:489–95. 10.3233/BMR-140545 25322741

[23] Binkley JM Stratford P Kirkpatrick S Farley CR Okoli J Gabram S. Estimating the reliability and validity of the upper extremity functional index in women after breast cancer surgery. Clin Breast Cancer. (2018) 18:e1261–7. 10.1016/j.clbc.2018.02.008 29551249

[24] Alnahdi AH Albarrati A. The upper extremity functional index: reliability and validity in patients with chronic obstructive pulmonary disease. Int J Environ Res Public Health. (2021) 18:10608. 10.3390/ijerph182010608 34682352 PMC8535980

[25] Kottner J Audige L Brorson S Donner A Gajewski B Hróbjartsson A. Guidelines for reporting reliability and agreement studies (grras) were proposed. Int J Nurs Stud. (2011) 48:661–71. 10.1016/j.ijnurstu.2011.01.016 21514934

[26] Beaton DE Bombardier C Guillemin F Ferraz MB. Guidelines for the process of cross-cultural adaptation of self-report measures. Spine. (2000) 25:3186–91. 10.1097/00007632-200012150-00014 11124735

[27] Hodkinson HM. Evaluation of a mental test score for assessment of mental impairment in the elderly. Age Ageing. (1972) 1:233–8. 10.1093/ageing/1.4.233 4669880

[28] Bujang MA Omar ED Baharum NA. A review on sample size determination for cronbach's alpha test: a simple guide for researchers. Malays J Med Sci. (2018) 25:85–99. 10.21315/mjms2018.25.6.9 30914882 PMC6422571

[29] Arafat SY Chowdhury HR Qusar M Hafez MA. Cross cultural adaptation and psychometric validation of research instruments: a methodological review. J Behav Health. (2016) 5:129–36. 10.5455/jbh.20160615121755

[30] Gladstone DJ Danells CJ Black SE. The fugl-meyer assessment of motor recovery after stroke: a critical review of its measurement properties. Neurorehabil Neural Repair. (2002) 16:232–40. 10.1177/154596802401105171 12234086

[31] Duncan PW Propst M Nelson SG. Reliability of the fugl-meyer assessment of sensorimotor recovery following cerebrovascular accident. Phys Ther. (1983) 63:1606–10. 10.1093/ptj/63.10.1606 6622535

[32] Edwards DF Lang CE Wagner JM Birkenmeier R Dromerick AW. An evaluation of the wolf motor function test in motor trials early after stroke. Arch Phys Med Rehabil. (2012) 93:660–8. 10.1016/j.apmr.2011.10.005 22336104

[33] Morris DM Uswatte G Crago JE Cook EW Taub E. The reliability of the wolf motor function test for assessing upper extremity function after stroke. Arch Phys Med Rehabil. (2001) 82:750–5. 10.1053/apmr.2001.23183 11387578

[34] Cederberg KLJ Sikes EM Bartolucci AA Motl RW. Walking endurance in multiple sclerosis: meta-analysis of 6-min walk test performance. Gait Posture. (2019) 73:147–53. 10.1016/j.gaitpost.2019.07.125 31326830

[35] Macchiavelli A Giffone A Ferrarello F Paci M. Reliability of the 6-min walk test in individuals with stroke: systematic review and meta-analysis. Neurol Sci. (2021) 42:81–7. 10.1007/s10072-020-04829-0 33064231

[36] Liu J Drutz C Kumar R McVicar L Weinberger R Brooks D. Use of the 6-min walk test post-stroke: is there a practice effect? Arch Phys Med Rehabil. (2008) 89:1686–92. 10.1016/j.apmr.2008.02.026 18760152

[37] Mak MK Lau AL Law FS Cheung CC Wong IS. Validation of the Chinese translated activities-specific balance confidence scale. Arch Phys Med Rehabil. (2007) 88:496–503. 10.1016/j.apmr.2007.01.018 17398252

[38] Botner EM Miller WC Eng JJ. Measurement properties of the activities-specific balance confidence scale among individuals with stroke. Disabil Rehabil. (2005) 27:156–63. 10.1080/09638280400008982 15824045

[39] Graf C. The Lawton instrumental activities of daily living scale. Am J Nurs. (2008) 108:52–62. 10.1097/01.NAJ.0000314810.46029.74 18367931

[40] Lachman ME Howland J Tennstedt S Jette A Assmann S Peterson EW. Fear of falling and activity restriction: the survey of activities and fear of falling in the elderly (safe). J Gerontol B Psychol Sci Soc Sci. (1998) 53:P43–50. 10.1093/geronb/53B.1.P43 9469171

[41] Chou KL Yeung FK Wong EC. Fear of falling and depressive symptoms in Chinese elderly living in nursing homes: fall efficacy and activity level as mediator or moderator? Aging Ment Health. (2005) 9:255–61. 10.1080/13607860500114035 16019279

[42] Hornyak V Brach JS Wert DM Hile E Studenski S VanSwearingen JM. What is the relation between fear of falling and physical activity in older adults? Arch Phys Med Rehabil. (2013) 94:2529–34. 10.1016/j.apmr.2013.06.013 23816923 PMC4878685

[43] Duncan PW Wallace D Lai SM Johnson D Embretson S Laster LJ. The stroke impact scale version 20 Evaluation of reliability, validity, and sensitivity to change. Stroke. (1999) 30:2131–40. 10.1161/01.STR.30.10.2131 10512918

[44] Liu TW Ng SS Ng GY. Translation and initial validation of the Chinese (Cantonese) version of community integration measure for use in patients with chronic stroke. Biomed Res Int. (2014) 2014:623836. 10.1155/2014/623836 24995317 PMC4065661

[45] Shrout PE Fleiss JL. Intraclass correlations: uses in assessing rater reliability. Psychol Bull. (1979) 86:420–8. 10.1037/0033-2909.86.2.420 18839484

[46] Koo TK Li MY. A guideline of selecting and reporting intraclass correlation coefficients for reliability research. J Chiropr Med. (2016) 15:155–63. 10.1016/j.jcm.2016.02.012 27330520 PMC4913118

[47] Pereira GS Silva SM Júlio CE Thonnard J Bouffioulx E Corrêa JCF. Translation and cross-cultural adaptation of satis-stroke for use in Brazil: a satisfaction measure of activities and participation in stroke survivors. Biomed Res Int. (2019) 2019:8054640. 10.1155/2019/8054640 30906780 PMC6398040

[48] Ostelo RW de Vet HC Knol DL van den Brandt PA. 24-item roland-morris disability questionnaire was preferred out of six functional status questionnaires for post-lumbar disc surgery. J Clin Epidemiol. (2004) 57:268–76. 10.1016/j.jclinepi.2003.09.005 15066687

[49] Stratford PW. Estimating the standard error of measurement from reliability studies. Physiother Can. (2004) 56:27–30. 10.2310/6640.2004.15377

[50] Haley SM Fragala-Pinkham MA. Interpreting change scores of tests and measures used in physical therapy. Phys Ther. (2006) 86:735–43. 10.1093/ptj/86.5.735 16649896

[51] Portney LG Watkins MP. Foundations of Clinical Research: Applications to Practice. London: Pearsons (2013).

[52] Ng SSM Liu TW Chan CY Chan ICW Chu JCL Poon HCH. Reliability and validity of the long-distance corridor walk among stroke survivors. J Rehabil Med. (2020) 52:jrm00062. 10.2340/16501977-2691 32412645

[53] Floyd FJ Widaman KF Butcher JN. Factor analysis in the development and refinement of clinical assessment instruments. Psychol Assess. (1995) 7:286–99. 10.1037/1040-3590.7.3.286

[54] Babyak MA Green SB. Confirmatory factor analysis: An introduction for psychosomatic medicine researchers. Psychosom Med. (2010) 72:587–97. 10.1097/PSY.0b013e3181de3f8a 20467001

[55] Brincks J Jorgensen JB Giese IE Palle ML Callesen J Johnsen E. A study of the discriminative properties of the six-spot step test in people with Parkinson's disease at the risk of falling. NeuroRehabilitation. (2019) 45:265–72. 10.3233/NRE-192801 31498144

[56] Liu TW Ng SSM Tan MP. Assessing the fall risks of community-dwelling stroke survivors using the short-form physiological profile assessment (s-ppa). PLoS ONE. (2019) 14:e0216769. 10.1371/journal.pone.0216769 31112580 PMC6528978

[57] Ng SS Shepherd RB. Weakness in patients with stroke: Implications for strength training in neurorehabilitation. Phys Ther Rev. (2000) 5:227–38. 10.1179/10833190078616665031250663

[58] Gemperline JJ Allen S Walk D Rymer WZ. Characteristics of motor unit discharge in subjects with hemiparesis. Muscle Nerve. (1995) 18:1101–14. 10.1002/mus.880181006 7659104

[59] Horstman AM Beltman MJ Gerrits KH Koppe P Janssen TW Elich P. Intrinsic muscle strength and voluntary activation of both lower limbs and functional performance after stroke. Clin Physiol Funct Imag. (2008) 28:251–61. 10.1111/j.1475-097X.2008.00802.x 18355344

[60] Lundquist CB Maribo T. The fugl-meyer assessment of the upper extremity: reliability, responsiveness and validity of the Danish version. Disabil Rehabil. (2017) 39:934–9. 10.3109/09638288.2016.1163422 27062881

[61] Ng SSM Liu TW Tsoh J Chen PM Cheng TS Cheung MCH. Psychometric properties of the trail walking test for people with stroke. Front Neurol. (2022) 13:821670. 10.3389/fneur.2022.821670 35309555 PMC8929289

[62] Gall S Phan H Madsen TE Reeves M Rist P Jimenez M. Focused update of sex differences in patient reported outcome measures after stroke. Stroke. (2018) 49:531–5. 10.1161/STROKEAHA.117.018417 29438087

